# Insomnia in Pregnancy and Factors Related to Insomnia

**DOI:** 10.1100/2012/197093

**Published:** 2012-04-24

**Authors:** Aynur Kızılırmak, Sermin Timur, Bahtışen Kartal

**Affiliations:** ^1^Semra ve Vefa Küçük Health College, Nevşehir Unıversity, Nevşehir, Turkey; ^2^Health College, Inonu University, 441280 Malatya, Turkey

## Abstract

This study aims to investigate insomnia experienced by pregnant women and factors associated with it. This study was designed as hospital-based, descriptive, and cross-sectional research. The participants were 486 people chosen with nonprobability random sampling method. The data were collected through Women's Health Initiative Insomnia Rating Scale, Beck Depression Inventory, and Interview Form. Insomnia prevalence in women participating in this study was found 52.2%. The results of logistic regression analysis showed that the risk of insomnia was 2.03 times higher for those in the third trimester than those in the first and second trimesters, 2.19 times higher for those 20 years old and over than younger ones, and 2.63 times higher for those who had depression syndrome than those who did not. Insomnia in pregnant women who participated in this study was found to be at high rates.

## 1. Introduction

Sleep is a physiological need for all human beings. Therefore, it is regarded as a prominent health variable that affects quality of life and wellness [1]. Sleep is necessary both for physical and psychological health. Healthy adults need to fall asleep in 5–10 minutes after they switch off the light and sleep for at least 7 hours [2, 3]. One of the prominent factors affecting sleep is different periods of life [4].

Pregnancy is one of the most important periods in women life. Despite being a natural phenomenon, pregnancy brings along major physiological, psychological, and social changes [5, 6]. Insomnia is one of the major problems experienced in pregnancy. In their study conducted with pregnant women, Çoban and Yanikkerem (2010) found that quality of sleep was deteriorated during pregnancy, and it decreased with the increasing gestational week [7]. Hedman et al. detected mean sleep duration as 8.7 in the first trimester while it was 8.4 and 8.3 for the second and third trimesters, respectively [8]. Insomnia in pregnancy might be caused by physical illnesses (nausea, backache, and frequent visits to the toilet), hormonal changes, growth of fetus, and inadequate respiration [9]. Besides, women can experience insomnia throughout pregnancy depending on the body position and increase in their abdomen size [10]. Insomnia, which causes deterioration in quality of life, becomes an important problem in pregnancy both for maternal and fetus health. Therefore, this study aims to investigate insomnia experienced by pregnant women and factors associated with it.

## 2. Methods

### 2.1. Design

This study was designed as a descriptive and cross-sectional research to investigate insomnia experienced by pregnant women and factors associated with it.

### 2.2. Participants

The study was conducted with the pregnant women who consulted to the gynecology policlinics in Aksaray Şambaz Vehbi Ekecik Maternity Hospital and the pregnancy unit in Nevşehir İ. Şevki Atasağun state hospital between August and December 2010.

The target population of the study is the pregnant women who consulted to the pregnancy units of the abovementioned hospitals during the study period. The participants were 486 pregnant women who were chosen from the target population using nonprobability random sampling method. The inclusion criteria were having no multiple pregnancies or any disease (heart disease, diabetes, etc.), being literate, and having no communication problems. The exclusion criteria were having such health problems as diabetes or hypertension during pregnancy, having communication problems and multiple pregnancies. Those who accepted to participate in the study but did not complete answering the questions were excluded from the study. Five of the women did not accept to participate in the study. The forms were piloted with 15 pregnant women to evaluate the comprehensibility of the questions. There are 7 gynecology policlinics in Şambaz Vehbi Ekecik Maternity hospital and each policlinic examines 15–20 pregnant women a day on average. However, as there is only one pregnancy unit, İ. Şevki Atasağun state hospital examines 60–70 pregnant a day women on average.

### 2.3. Data Collection and Instruments

The data were collected from those who met the research criteria and were examined in the polyclinics. The data were collected on four weekdays every week. Considering that the participants would be more relaxed, the data were collected after examination. The data collection forms were filled in by the researcher herself during the face-to-face interviews conducted with those volunteering to participate in the study.

Data were collected using the Interview Form (IF), the Women's Health Initiative Insomnia Rating Scale (WHIIRS), and the Beck Depression Inventory (BDI). The IF was developed on a literature basis by the investigators [8, 11, 12].

#### 2.3.1. Interview Form

The form consists of questions related to the descriptive characteristics of women (age, educational status), features related to fertility (number of pregnancies, gestational week), and sleeping habits of the participants (average sleeping hour, differences in sleep before and after pregnancy, taking precautions for insomnia, having snoring habit or not). Those who reported to smoke at least one cigarette were identified as “smokers.” The amount of tea/coffee consumed daily was identified according to the number of tea glasses for tea and number of coffee cups for coffee.

#### 2.3.2. Women's Health Initiative Insomnia Rating Scale (WHIIRS)

Women's Health Initiative Group used a scale consisting of 10 items to evaluate insomnia on 66.269 postmenopausal women aged between 50 and 79 years. Levine et al. developed Women's Health Initiative Insomnia Rating Scale (WHIIRS) by creating 5 questions with these ten items [13–15]. The reliability and validity of the scale were enhanced by Timur and Sahin for Turkey [16]. The scale is a 5-point Likert type in which the first 4 questions aim to identify the beginning of insomnia, sleep-maintenance insomnia, and the state of waking up early in the mornings, while the last question is associated with quality of sleep. Each question was replied considering the women's experience in the last 4 weeks and the frequency of the experience in each week. The first four questions in WHIIRS are rated between 0 and 4 and evaluated depending on the answers. “0” score indicates that there is no problem in relation to insomnia, while “4” indicates that there are problems in relation to insomnia for 5 or more times in a week. The highest score obtained from the scale demonstrated the highest levels of insomnia symptoms. The scores ranged between 0 and 20. The Cronbach Alpha value of the WHIIRS was found 0.85 by Timur and Sahin (2009) [13–16]. The reliability of the scale was analyzed in this study as well, and Cronbach Alpha value was found 0.77, which indicated its reliability and validity.

#### 2.3.3. Beck Depression Inventory (BDI)

Level of depression was measured using the BDI. BDI was developed by Beck et al. (1974) and adapted to Turkish by Hisli (1988). The BDI is a self-report scale with 21 items that measure the emotional, somatic, cognitive, and motivational symptoms seen in the individual with depression. The aim of the scale is not to diagnose depression, but to objectively determine the severity of depressive symptoms. BDI scores greater than or equal to 17 have been reported to indicate depression with more than 90% accuracy that might require treatment. The score range on each item is 0–3, and the overall depression score is simply the sum of the item scores. The highest score is 63 [17, 18]. In the samples of this study, Cronbach's alpha value was 0.83. 

### 2.4. Statistical Analysis

The data obtained from the study were analyzed using SPSS for Windows 10.0 package programming through one way Anova test (ANOVA), significance test between the two mean scores (*t*), and Backward Stepwise Logistic Regression Analysis. Measurable data were presented together with means $\left(\bar{X}\right)$ and Standard Deviations (SD). Variables that affect insomnia were identified according to the Backward Stepwise Logistic Regression analysis results, and statistical significance was taken as *P* < 0.05 [19].

### 2.5. Ethical Considerations

Before the study, written consent was obtained from the home institution of the hospitals where the study would be conducted. Besides, the participants were informed about the research and assured that their personal information would be kept confidential. They were also told that they could withdraw from the study any time they want, and those who volunteered were involved in the study.

## 3. Results

Average age of the participants is 25.2 ± 5.49 years (Min: 15, Max: 42), and 78.4% of them are over 20 years old. Besides, 89.5% of the pregnant women are housewives, 44.2% of them are illiterate/literate or primary school graduate, and 33.3% of them had low economic income.

Table 1 shows the distribution of some characteristics of the participants. It was found that 57.8% of the participants were in the third trimester, while 30.3% of them were in the second trimester. Besides, 58% of them used medicine, and 58.2% of them were with a body mass index (BMI) of 25 and over. It was found that 24.9% of the pregnant women had depression symptoms, 4.1% smoked, 80% drank 2 or more glasses of tea a day, but 81.1% reported that they never drank coffee. 

Table 2 shows the distribution of pregnant women according to their sleep habits. The average sleep duration was detected 7 ± 2.26 h (first trimester: 8.3 ± 2.0, 2 daily, second trimester: 8.1 ± 2.1, third trimester: 7.3 ± 2.3) (*P* < 0.001). Seventy-one percent of the participants reported that their sleep habits changed in pregnancy, and 61.7% of them said that they began to sleep earlier than before. Besides, 51.2% reportedly had insomnia and 10.1% had snoring habit. Reasons for their insomnia according to the participants were, respectively, frequent visits to the toilet (39.3%), not finding a comfortable position while sleeping (30.7%), and restless legs (13.7%).

Table 3 shows the distribution of WHIIRS average scores according to some characteristics of the participants. WHIIRS mean for those over 20 years of age was detected 8.4 ± 5.06, while it was found 7.0 ± 4.43 for those under 20 years of age, and the difference was found statistically significant (*P* < 0.05). WHIIRS average score for those whose education level was illiterate/literate/primary school graduate was 8.6 ± 4.99, while it was 7.7 ± 4.92 for those who are high school or university graduates. The difference was found statistically significant (*P* < 0.05). The mean score of 6.4 ± 5.14 in the first trimester was found to increase up to 9.2 ± 4.95 in the third trimester. The difference between the scores was found significant.

WHIIRS mean score of those whose BDI scores were 17 and over was found 10.7 ± 5.09, while it was 7.3 ± 4.63 for those whose BDI score was under 17. The difference was found to be statistically significant (*P* < 0.005). The difference between WHIIRS scores for those who used medicine (8.6 ± 4.85) and who did not (7.5 ± 5.06) was also found significant (*P* < 0.005). The present study shows that WHIIRS scores increase in parallel with the gestational week. WHIIRS mean scores of women with BMI of 25 and over (10.7 ± 5.09) were found significantly higher than those with BMI of under 25 (*P* < 0.05).

No significant relationship was detected between WHIIRS scores and smoking and drinking tea or coffee (*P* < 0.05).

Table 4 presents the Backward Stepwise Logistic Regression analysis results in the model created according to bivariate analysis results which detected a significant relationship between depression probability and variables such as age, education level, BMI, BDI, existence of psychiatric disease, used medicine, and gestational trimester. Analysis results show that being in the third trimester (OR: 2.03), being 20 years old or older (OR: 2.19), and having BDI over 17 (OR: 2.63) were found to be prominent risk factors for insomnia.

## 4. Discussion

Average sleep duration of the pregnant women in this study is 7.7 ± 2.26 h. Sleep experts indicate that quality of sleep is more important than its duration [20]. Despite the fact that average daily sleep duration of the pregnant women in this study was found to be in normal standards, more than half of the participants reported to have insomnia, which indicates their low quality of sleep. Lee et al. (2000) found an increase in the sleep duration, but a decrease in sleep quality in the first trimester [21]. Findings of this study are compatible with those in the literature.

The present study found that sleep duration decreased with the increase in the gestational trimester, and there was an increase in the WHIIRS mean scores of the participants. Logistic regression results show that the risk of developing insomnia in those in the third trimester is 2.03 times more than those in other trimesters. Lee (1999) found that women in the third gestational week had sleep problems two times more than before [22]. The related literature shows that insomnia is more common in the third trimester when the problems actually result from physical and psychosocial changes [8, 12, 23–25]. Besides, the reasons for insomnia detected in this study (frequent visits to the toilet, not finding a comfortable position while sleeping, and restless legs) were more common in the last trimester [8, 26, 27]. Therefore, those in the third trimester are believed to have insomnia more often than those in other trimesters. Findings of this study are correlated with the literature.

Three out of each four participants reported that their sleep habits changed in pregnancy and more than half of them stated that they began to sleep less than before. Hedman et al. [8] and Kennelly et al. [23] found that sleep duration increased in the first gestational trimester, while there was a decrease in the other trimesters. Findings of this study in relation to sleep duration according to gestational trimester differ from those of Hedman et al. [8] and Kennelly et al. [23]. This difference could result from the investigation of changes in sleep habits in different trimesters of pregnancy.

Insomnia is one of the prominent problems in pregnancy. It was found that more than half of the pregnant women participating in this study experienced insomnia. The related literature demonstrates that the complaint of insomnia was reported by more than half of the women in the third trimester [28, 29]. Similarly, Çoban and Yanikkerem (2010) found that more than half of the women (54%) stated that they had bad sleep quality in pregnancy [7]. Findings of this study are compatible with those in the literature.

During pregnancy, many steroid hormones including progesterone, estrogen, and prolactin are secreted from placenta. Among these hormones, progesterone has certain side-effects.In addition, progesterone also has certain effects like inhibitor on smooth muscles and nervous system, and it also affects respiration, and breathing becomes shallow. Sleep problems during pregnancy could also be caused by insomnia due to progesterone, frequent visits to the toilet, shortness of breathing, nausea, vomiting, problems related to other gastrointestinal system, hormonal changes, and growing fetus [9, 30]. Hedman et al. (2002) detected some of the reasons for insomnia in pregnancy as growth of fetus and not finding a comfortable position while sleeping [8]. In line with the literature, this study found the reasons for insomnia in pregnant women as frequent visits to the toilet, not finding a comfortable position while sleeping, and restless legs, respectively.

Another very common problem experienced in pregnancy is snoring. Snoring in pregnancy can be repetitive, loud, and increase gradually [28–31]. It was found that one out of every ten women participating in this study had snoring habit. Guilleminault et al. (2000) found that while the frequency of snoring before pregnancy was 3.7%, it increased up to 11.8% in the sixth month of pregnancy [29]. Increase in snoring habit was reported in other studies as well [8, 31–33]. Findings of this study in relation to snoring are compatible with those in the literature. However, this study did not investigate snoring habits before and after pregnancy.

Total sleep duration gradually decreases with aging [20, 34, 35]. It was found that insomnia in pregnancy increased with age. Pregnant women who were 20 years old and over reported that their insomnia increased 2.1 times during pregnancy.

This study also detected that the participants who had depression symptoms had the risk of developing insomnia 2.6 times more than those who did not. Several studies in the literature point the highly significant relationship between depression and insomnia [36–38]. Findings obtained from this study are compatible with those of in the literature.

It was found that pregnant women with low education levels experienced insomnia more frequently, but the logistic regression analysis did not detect education as a risk factor for insomnia in pregnancy. Çoban and Yanıkkerem (2010) did not detect a relationship between education level and quality of sleep in pregnant women, either [7]. Findings of this study are compatible with those of Çoban and Yanıkkerem.

No significant relationship was detected between the state of smoking and insomnia. Kaneita et al. (2005) identified that smoking pregnant women experienced sleep disturbances 1.4 times more frequently than those who did not [39]. Findings of this study are different from those of Kaneita et al. (2005). The difference is assumed to result from the low percentage of smoking women in this study (4.1%).

Caffeine reduces total sleep duration and increases the duration to fall asleep. However, these effects can be experienced when these drinks are consumed late at night or more than 500 mg in a day [1, 20, 40]. This study detected no relationship between drinking tea-coffee and insomnia symptoms. The difference is assumed to result from the facts that the great majority of the participants did not drink coffee (81%) and despite the great majority reported to consume tea (80% ≥2 tea glasses), the tea did not contain too much caffeine.

WHIIRS mean scores were found to be higher in women with BMI of 25 and over. Although the findings of this study also found that women with BMI of 25 and over had more complaints about insomnia, logistic regression analysis did not detect BMI as a risk factor. Kennelly et al. (2011) investigated the effect of BMI on insomnia in pregnancy and found no relationship between sleep habit and BMI [23]. Findings of this study are compatible with those of Kennelly et al. (2011).

More than half of the participants in this study reported to have insomnia. Although the sleep duration of the pregnant women was found within normal standards, it was detected to decrease with the increasing gestational trimester. This study identified age, depression symptoms, and gestational trimester as risk factors for insomnia. In line with these findings, it is recommended that health professionals should evaluate complaints about insomnia in pregnancy, suggest protective and supportive care, and direct pregnant women with early diagnosis and treatment.

## Figures and Tables

**Table 1:** Distribution of some of the characteristics of the participants (*N* : 486).

Fertility characteristics	*N*	%	$\bar{X}$ ± SD
Gestational trimester			
First	58	11.9	27.5 ± 10.13
Second	147	30.3	
Third	281	57.8	

Used medicine			
^&^Present	282	58.0	
Not present	204	42.0	

**^+^**BMI (Kg/m^2^)			
<25	203	41.8	26.1 ± 4.20
≥25	283	58.2	

*BDI score			
≥17	121	24.9	12.7 ± 7.78
<17	365	75.1	

Smoking in pregnancy			
Yes	20	4.1	
No	451	92.8	
Quitting smoking in pregnancy	15	3.1	

Drinking tea/glass			
No	41	8.5	
1	56	11.5	3.2 ± 2.4
≥2	389	80.0	

Drinking coffee/coffee cup			
No	394	81.1	
1	69	14.2	1.0 ± 1.4
≥2	23	4.7	

*BDI: Beck Depression Inventory (Min: 0, Max: 45).

^+^BMI: Body Mass Index.

^&^Iron, multivitamin.

**Table 2:** Distribution of the pregnant women according to their sleep habits (*N* : 486).

Sleep habits	$\bar{X}$± SD	
^&^Total sleep hours (daily)	7.7 ± 2.26	
First trimester	8.3 ± 2.0	
Second trimester	8.1 ± 2.1	
Third trimester	7.3 ± 2.3	

Amount of sleep needed now compared to before	*N*	%
There is a difference	345	71.0
There is no difference	141	29.0

Change in sleep habit (*n* : 345)		
More in the past—less at the present	213	61.7
Less in the past—more at the present	132	38.3

Insomnia		
Yes	249	51.2
No	237	48.8

Habitual snoring		
Yes	49	10.1
No	437	89.9

*Reason for insomnia (*n* : 459)		
Frequent visits to the toilet	184	39.3
Not finding a comfortable position while sleeping	150	30.7
Restless legs	67	13.7
Psychological problems	19	4.2
Medicine used	9	1.8
Being alone at home	9	1.8
Other	21	4.3

^+^Taking precaution for insomnia (*n* : 249)		
Yes	92	36.9
No	157	63.1

*More than one answer is given.

^&^ *F*: one-way ANOVA test (*P* < 0.001).

^+^Drinking milk, bathing.

**Table 3:** The distribution of Women's Health Initiative Insomnia scale scores according to some characteristics of the participants.

Age	WHIIRS $\bar{X}\;$± SD	Test and significance
<20	7.0 ± 4.43	*t* : −2.734
≥20	8.4 ± 5.06	*P* : 0.006

Education level		
Illiterate/literate/primary school	8.6 ± 4.99	*t* : 2.049
High school/university	7.7 ± 4.92	*P* : 0.041

Gestational trimester		
First	6.4 ± 5.14	*F* : 16.112
Second	6.8 ± 4.41	*P* : 0.001
*Third	9.2 ± 4.95	

^&^BDI score		
≥17	10.7 ± 5.09	*t* : 6.840
<17	7.3 ± 4.63	*P* : 0.001

Used medicine		
Present	8.6 ± 4.85	*t* : 2.529
Not present	7.5 ± 5.06	*P* : 0.012

Smoking in pregnancy		
Yes	8.1 ± 5.0	*F* : 0.846
No	7.9 ± 3.73	*P* : 0.430
Quitting smoking in pregnancy	9.8 ± 5.38	

Drinking coffee/coffee cup		
No	8.2 ± 4.9	*F* : 0.404
1	7.7 ± 4.9	*P* : 0.668
≥2	8.6 ± 5.7	

Drinking tea/glass		
No	8.4 ± 5.36	*F* : 2.045
1	6.9 ± 5.03	*P* : 0.130
≥2	8.3 ± 4.90	

^+^BMI (Kg/m^2^)		
<25	7.4 ± 4.73	*t* : −2.603
≥25	8.6 ± 5.08	*P* : 0.01

Total	8.1 ± 4.97	

*The group making difference.

*t*: Student *t* test.

*F*: one-way ANOVA test.

^&^BDI: Beck Depression Inventory.

^+^BMI: Body Mass Index.

**Table 4:** Advanced analysis of the risk factors associated with insomnia in pregnant women*.

Risk factors for insomnia	*β*	SE^a^	df^b^	*P*	OR^c^	95% Cl^d^	
Gestational trimester							
1st	(Reference)						
2nd	0.257	0.327		0.432	1.29	0.681–2.456	
3rd	0.711	0.306	2	0.020	2.03	1.117–3.708	

Age							
<20	(Reference)						
≥20	0.775	0.235	1	0.001	2.17	1.370–3.442	

^&^BDI score							
<17	(Reference)						
≥17	0.969	0.228	1	0.000	2.63	1.684–4.121	

*Backward stepwise logistic regression.

^&^BDI: Beck Depression Inventory.

SE^a^: standard error; df^b^: degree of freedom; OR^c^: odd's ratio; Cl^d^: confidence interval.
